# (DA-U)^2^Net: double attention U^2^Net for retinal vessel segmentation

**DOI:** 10.1186/s12886-025-03908-0

**Published:** 2025-02-21

**Authors:** Bing Chu, Jinsong Zhao, Wenqiang Zheng, Zhengyuan Xu

**Affiliations:** 1 Department of Medical Engineering, Wannan Medical College, WuHu, AnHui 241002 China; 2 School of Medical Imageology, Wannan Medical College, WuHu, AnHui 241002 China; 3 Department of Nuclear Medicine, First Affiliated Hospital of Wannan Medical College, Wuhu, AnHui 241001 China

**Keywords:** Feature Channel Attention Mechanism, CBAM, Deep-Learning, Retinal Segmentation

## Abstract

**Background:**

Morphological changes in the retina are crucial and serve as valuable references in the clinical diagnosis of ophthalmic and cardiovascular diseases. However, the retinal vascular structure is complex, making manual segmentation time-consuming and labor-intensive.

**Methods:**

This paper proposes a retinal segmentation network that integrates feature channel attention and the Convolutional Block Attention Module (CBAM) attention within the U^**2**^Net model. First, a feature channel attention module is introduced into the RSU (Residual Spatial Unit) block of U^**2**^Net, forming an Attention-RSU block, which focuses more on significant areas during feature extraction and suppresses the influence of noise; Second, a Spatial Attention Module (SAM) is introduced into the high-resolution module of Attention-RSU to enrich feature extraction from both spatial and channel dimensions, and a Channel Attention Module (CAM) is integrated into the lowresolution module of Attention-RSU, which uses dual channel attention to reduce detail loss.Finally, dilated convolution is applied during the upscaling and downscaling processes to expand the receptive field in low-resolution states, allowing the model to better integrate contextual information.

**Results:**

The evaluation across multiple clinical datasets demonstrated excellent performance on various metrics, with an accuracy (ACC) of 98.71%.

**Conclusion:**

The proposed Network is general enough and we believe it can be easily extended to other medical image segmentation tasks where large scale variation and complicated features are the main challenges.

## Introduction

The morphological attributes of the retinal vasculature at the fundus, such as shape, length, curvature, and branching, are clinically important distinguishing signs [1]. Clinicians can study changes in the body’s microvasculature on the basis of alterations in the morphology of the retinal vessels [2]. For example, the hardening, exudation, and constriction of retinal arterial vessels can reflect a trend toward higher blood pressure within the body [3]; conditions such as leakage, edema, and hemorrhage of the retinal vessels can serve as diagnostic criteria for diabetes and macular diseases [4–6]. The segmentation of retinal vessels is a necessary step to obtain the above critical information, and accurate segmentation results help doctors diagnose eye diseases precisely [7, 8]. Owing to the complex structure of retinal vessels, manual segmentation is time-consuming, labor-intensive, and subject to subjective factors, making automated segmentation via computer technology a popular topic in retinal segmentation.

With the continuous development of computer technology, its application in retinal segmentation has become a popular research topic among medical and computer science scholars domestically and internationally [9, 10]. Koukounis et al. used a matched filtering algorithm to increase the difference between vessels and the rest of the retina, thereby achieving retinal vessel segmentation [11]. However, some retinal vessels are small and dense, leading to detail loss with this algorithm. Fraz et al. used morphological reconstruction and Gabor filters for the multiscale localization and segmentation of retinal images [12]. However, morphology is prone to noise interference and binarization difficulties during operation. Improvements in hardware technology have enabled methods based on deep learning to overcome the limitations of traditional approaches, demonstrating powerful capabilities in image processing tasks. Medical image segmentation based on deep learning has become popular research direction in clinical diagnosis [13–16]. The Fully Convolutional Neural Network (FCN) was the first neural network for image semantic segmentation tasks, it uses convolutional layers instead of fully connected layers to achieve image segmentation [17, 18]. Ronneberger et al. proposed a U-shaped network structure, U-Net, consisting of an encoder composed of convolutional and pooling layers, and a decoder composed of upsampling and deconvolution layers, with skip connections added at corresponding levels of the U-structure to effectively merge low-level and high-level features [19]. It has been widely applied in the field of medical image segmentation and has achieved good results. The attention mechanism, inspired by the ability of humans to select attention points on the basis of voluntary and involuntary cues, focuses the model on information strongly related to the task and has been widely applied in various neural network models [20]. Li et al. embedded the attention module into the U-Net network structure for retinal vascular images, improving model accuracy [21].

However, owing to the irregular morphology, dense and disordered distribution, and unclear boundaries of retinal vessels, there is room for improvement in existing methods.To address issues such as incomplete extraction of the underlying features of the retina, susceptibility to background noise, and neglect of the relevance of contextual information in existing methods, this paper proposes a method that integrates feature channel attention mechanisms [22], the Convolutional Block Attention Module (CBAM) [23], and U^2^Net [24]. In summary, the contributions of this paper can be summarized as follows:

- The backbone network is U^2^Net, and feature channel attention mechanisms are integrated between decoders and encoders at corresponding levels of RSU (Residual U-Block, RSU) units in U^2^Net, with a focus on salient regions during the extraction of underlying features.
- During the sampling process at high-resolution levels, a feature space attention mechanism is introduced to form feature maps of dual importance in feature channels and feature space, enriching feature extraction from both channel and spatial dimensions. Feature channel attention mechanisms are again added to the output of the sampling process at low-resolution levels to form dual attention feature maps for fine areas, which focus more on detail feature information and suppress noise points.
- This achieves the capture of more contextual information from different scales, paying more attention to defect area features, suppressing background noise, reducing loss, and improving segmentation efficiency.

## Related Work

### U^2^Net

**Fig. 1:**
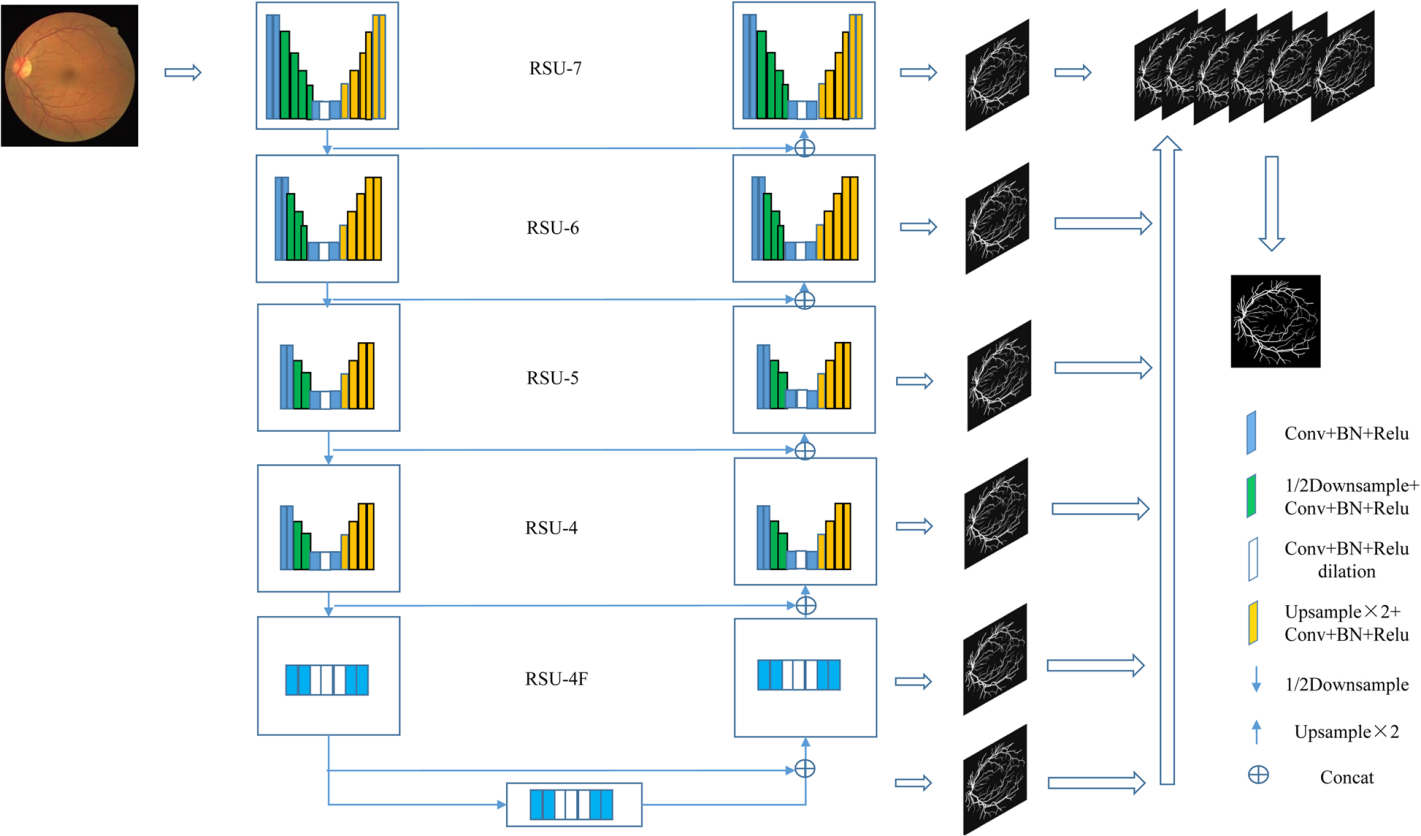
Architecture of the U^2^Net network

The U^2^Net network is a dual-layer nested network proposed by Qin et al. and is based on U-Net, which is primarily used for Salient Object Detection (SOD) tasks [25].As shown in Fig. 1.

U^2^Net has three advantages in image segmentation:

- Independent feature extraction by different levels of RSU modules, increasing the depth and breadth of complex image feature information extraction;
- Effective fusion of output information from different levels, enabling the model to better integrate contextual information and improve model accuracy;
- The model includes numerous pooling processes, preventing increased computational costs due to complex structures.

### Feature channel attention mechanism

The feature channel attention mechanism establishes interconnections between channels, acquires the importance coefficients of each channel through learning, and depends on these importance coefficients to enhance features with high coefficients while suppressing the expression of features with low coefficients.

**Fig. 2:**
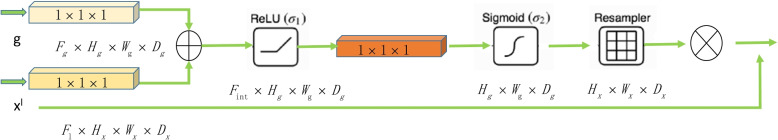
Architecture of feature channel attention

Its working principle is illustrated in Fig. 2. The feature inputs x^*l*^ and g undergo 1 × 1 convolution transformations to match channel numbers and are than added together. This is followed sequentially by the ReLU, a 1 × 1 convolution kernel, and the Sigmoid to obtain attention allocation coefficients between 0 and 1. These coefficients are then applied to various parts of the feature map. Finally, the initial input x^*l*^ is combined with the attention output map to produce the final output.

### CBAM attention module

**Fig. 3:**
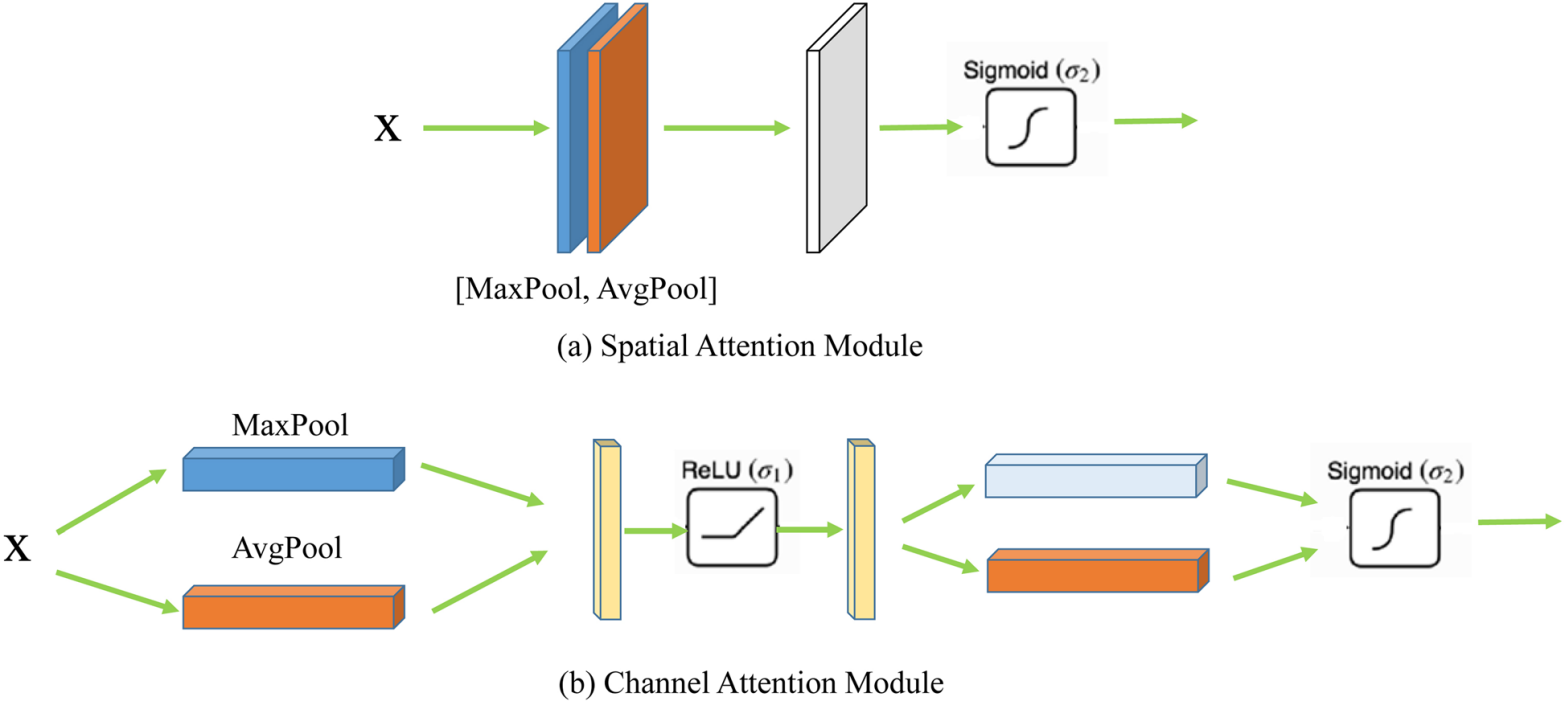
Architecture of the CBAM Attention Module: the CBAM contains two parts, the Spatial Attention Module (SAM) and the Channel Attention Module (CAM)

The Convolutional Block Attention Module (CBAM) comprises two parts: the Spatial Attention Module (SAM) and the Channel Attention Module (CAM). As shown in Fig. 3, the SAM module processes the input *X (H* × *W* × *D)* through mean pooling and max pooling to generate two feature maps of size *H* × *W* × *1*. These maps then go through a convolution calculation with a kernel size of 7 to produce a new feature map, which, after being a Sigmoid, results in the final feature map adjusted for spatial attention. The CAM module is similar to the previously described feature channel attention mechanism: it takes input *X* and applies mean pooling and max pooling, followed by two convolution operations and a ReLU to generate a new feature map that captures the importance coefficients between channels. This map is then concatenated with feature weights and processed through a sigmoid function to produce the final feature map adjusted for channel attention.

Within the CBAM attention mechanism, the spatial attention mechanism focuses on the positional information of targets, enhancing the spatial accuracy of segmentation tasks. Moreover, the channel attention mechanism, by leveraging both pooling methods, enriches the extraction of high-level features, thereby improving the accuracy of segmentation tasks in the feature dimension.

## Methodology

The structure of the retina is complex, contains a large amount of detailed feature information, and is susceptible to interference from background noise. Therefore, from a structural perspective, the network model needs to extract both high- and low-level feature information to the greatest extent possible, and functionally, it should focus on key parts within the feature information. Inspired by the rich feature extraction capabilities of the U^2^Net neural network and the principles of attention mechanisms, this paper proposes the (DA-U)^2^Net model, which is based on the main framework of U^2^Net and introduces attention mechanisms into the RSU (Residual U-Block) unit modules, thereby constructing a dual attention model.

### (DA-U)^2^Net

**Fig. 4:**
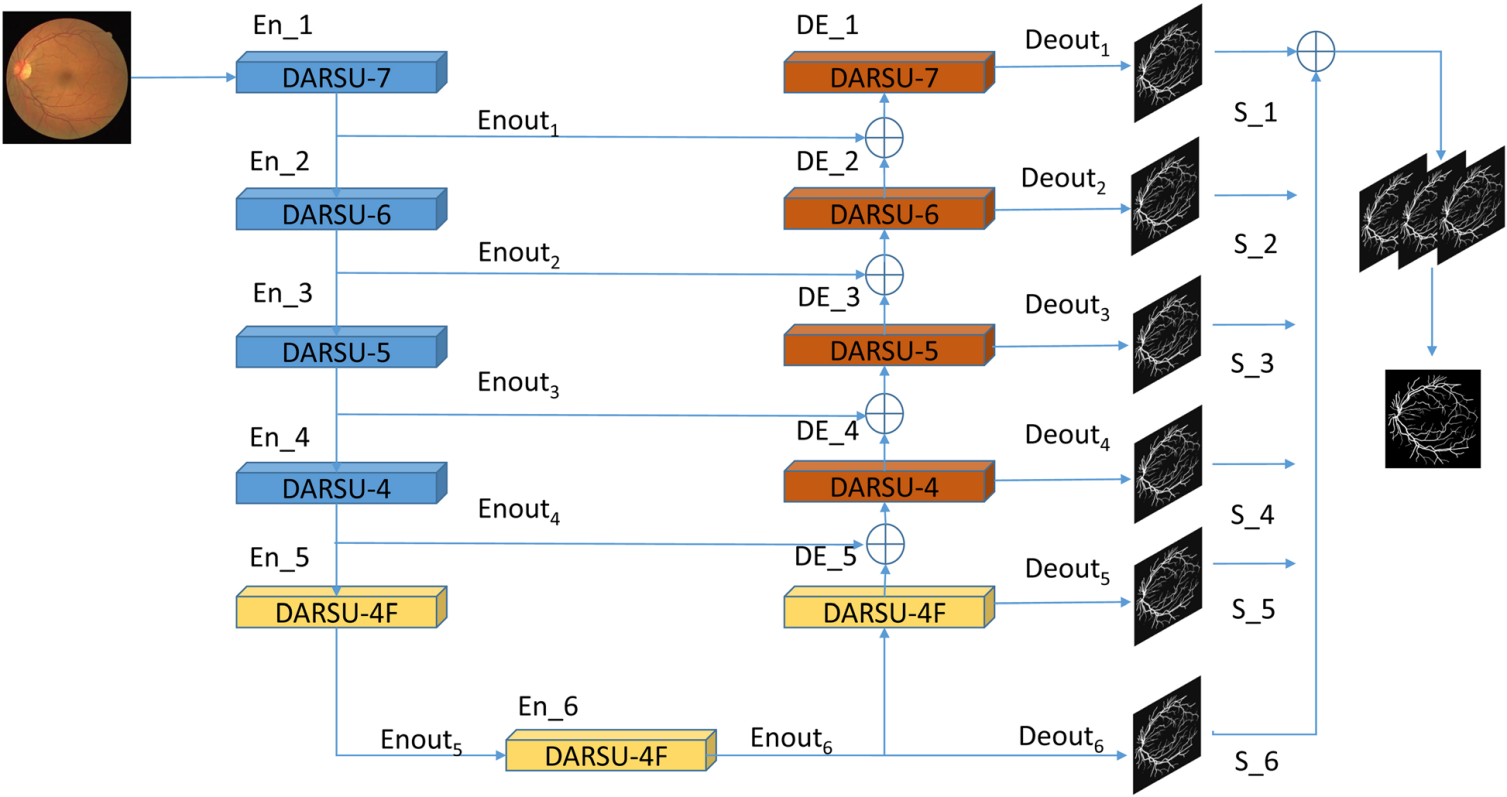
Architecture of the (DA-U)^2^Net network

As shown in Fig. 4, (DA-U)^2^Net includes six encoding parts: En_1, En_2, En_3, En_4, En_5, and En_6,and five decoding parts: De_1, De_2, De_3, De_4, and De_5. Among these, En_1 and De_1 utilize the DARSU-7 module;En_2 and De_2 use the DARSU-6 module; En_3 and De_3 use the DARSU-5 module; En_4 and De_4 use the DARSU-4 module; and En_5, En_6, and De_5 use the DARSU-4F module. Each DARSU module is a U-shaped network constructed by referring to U-Net and integrating two attention mechanisms at different levels, with the numbers 7, 6, 5, and 4 indicating the depth of the U-shaped network. As an example of DARSU-4F, it first undergoes three downsampling operations, followed by three upsampling operations. DARSU-5, DARSU-6, and DARSU-7 sequentially add one more upsampling and downsampling operation each. DARSU-4F is a special module; given the significantly reduced resolution of the image after multiple downsampling steps, continuing to downsample further decreases the image resolution, causing loss of detail information during the upsampling process. Therefore, this process does not follow the aforementioned pattern but instead uses dilated convolutions to replace the upsampling and downsampling processes. Dilated Convolution is a process that expands the convolutional kernel’s receptive field without changing the number of parameters, ensuring that the output feature map remains unchanged [26]. It can capture information from different ranges by adjusting the dilation rate. Finally, the outputs from each upsampling RSU module are fused with the final output of the U-structure to obtain the final prediction map.

### U channel and spatial attention module

In U^2^Net, the RSU-7, RSU-6, RSU-5, and RSU-4 modules effectively extract features at different levels for the task, but the abundance of feature information may cause the network to overlook significant regions. To retain effective feature information in the context and improve the model’s accuracy, a dual attention mechanism is introduced into these four modules.

**Fig. 5:**
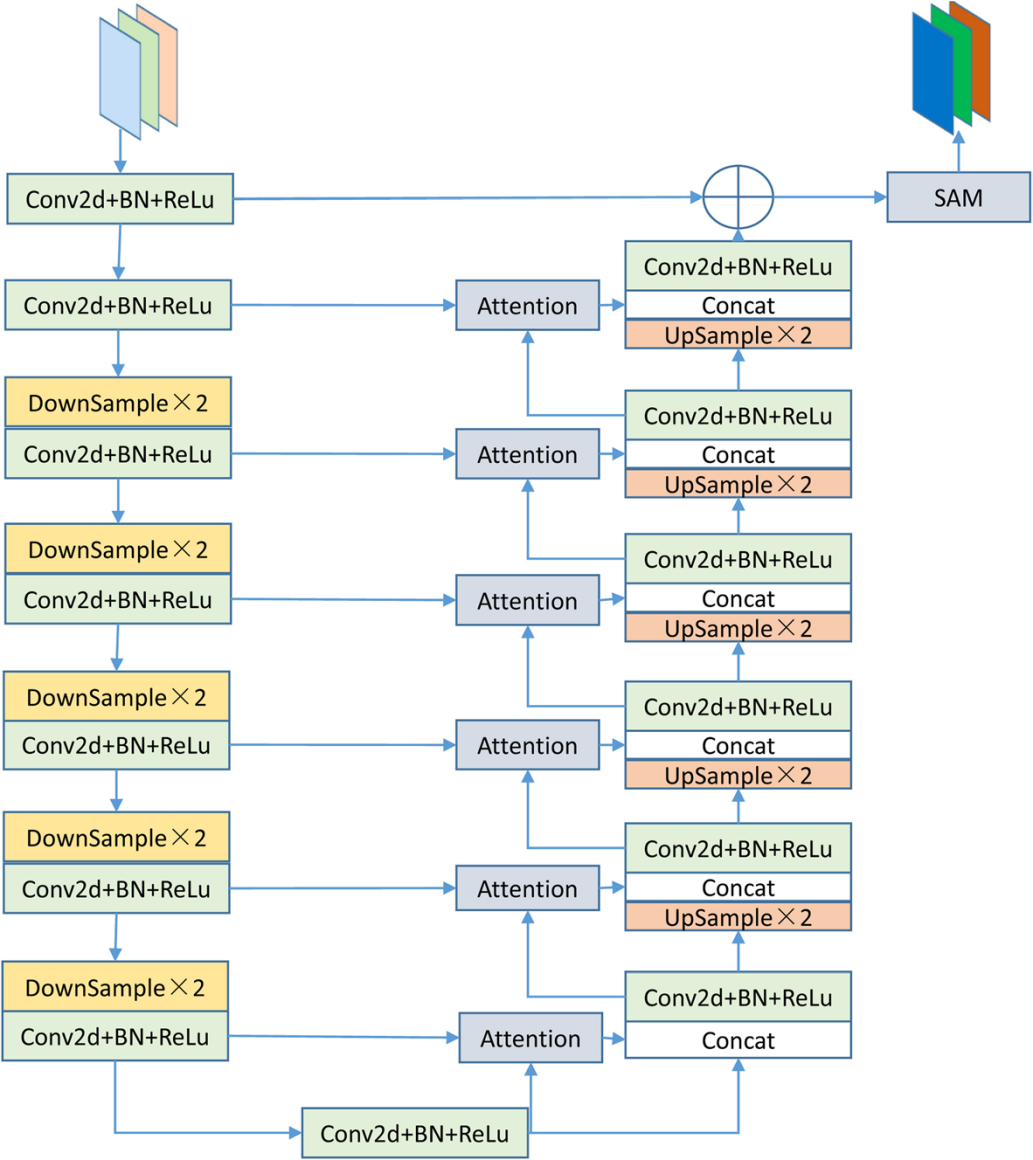
Architecture of the DARSU-7 module

As illustrated in Fig. 5, taking DARSU-7 (Double Attention Residual UBlock, DARSU) as an example, the remaining encoding part first goes through two 3 × 3 convolutions (Conv2d), normalization (BN), and ReLU, followed by five downsampling operations (2 × 2 max pooling, DownSample), each of which includes Conv2d + BN + ReLU. In the right decoding part, the result from the previous level is processed with the encoding part of the corresponding level through the feature channel attention mechanism, and the generated feature map is then concatenated with the result from the previous level, with each upsampling process also including Conv2d + BN + ReLU. The processed feature map extracts multiscale features and, by incorporating the attention mechanism, focuses more on significant features. It then sequentially encodes these into high-resolution feature maps, reducing the loss of detail features during the upsampling process. The final concatenated output is then fed into the SAM module of the CBAM, effectively enhancing the model’s sensitivity to spatial information features and balancing detail features with global spatial information features in high-resolution feature maps. DARSU-6, DARSU-5, and DARSU-4 all adopt the same architecture, except that the number of upsampling and downsampling layers decreases by one layer each.

**Fig. 6:**
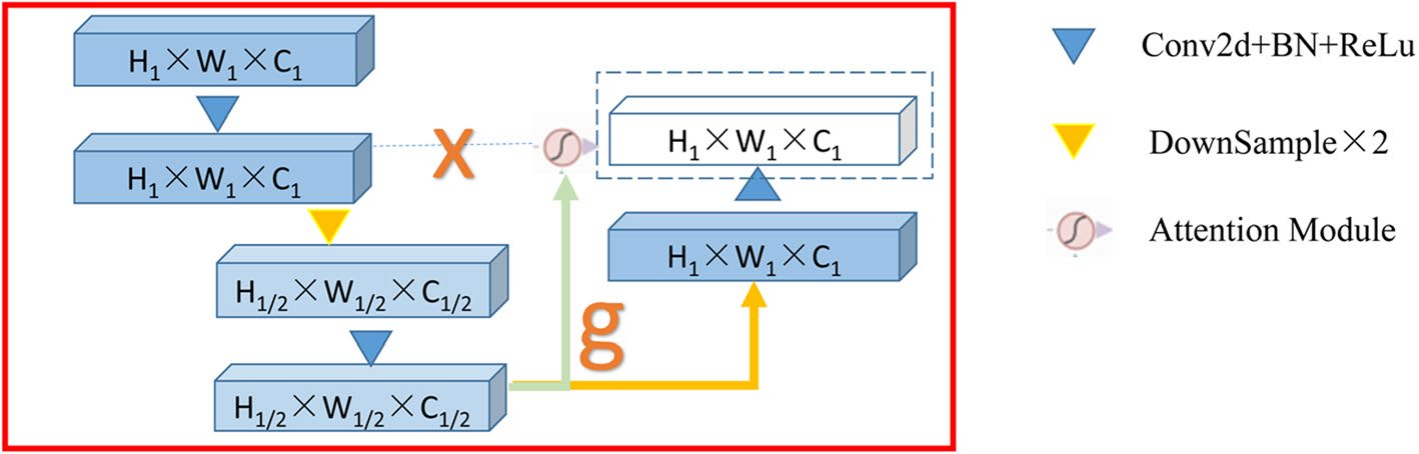
Attention mechanism structure in skip connections

In the aforementioned module, it is important to note the feature channel attention mechanism within the skip connections, as shown in Fig. 6. Let the feature map from the previous layer be *H*_1_ × *W*_1_ × *C*_1_, which after convolution becomes *x*, and after downsampling becomes half the original feature map size, *H*_1*/*2_ × *W*_1*/*2_ × *C*_1*/*2_. This, after convolution, results in *g*, which is first used as the feature input (as described in Sect. 2.2) together with the output *x* from the previous layer’s feature map through the feature channel attention mechanism to produce a new feature map. This new map is then concatenated with the upsampled result of *g*, *H*_1_ × *W*_1_ × *C*_1_, and serves as the input for the next layer. This process not only better integrates contextual information into the new feature map but also suppresses the expression of features in less important regions.

The input to the module is defined as *g*, with *x* representing the skip connection coming from the expansion path. The computation formula is as follows:

1 $$x_{attention} = Attention(g,x)$$

2 $$x_{upsampled} = Upsample(g,scale = 2)$$

3 $$output = ConvBlock(concatenate(x_{attention} ,x_{upsampled} ))$$

### U channel dual attention module

**Fig. 7:**
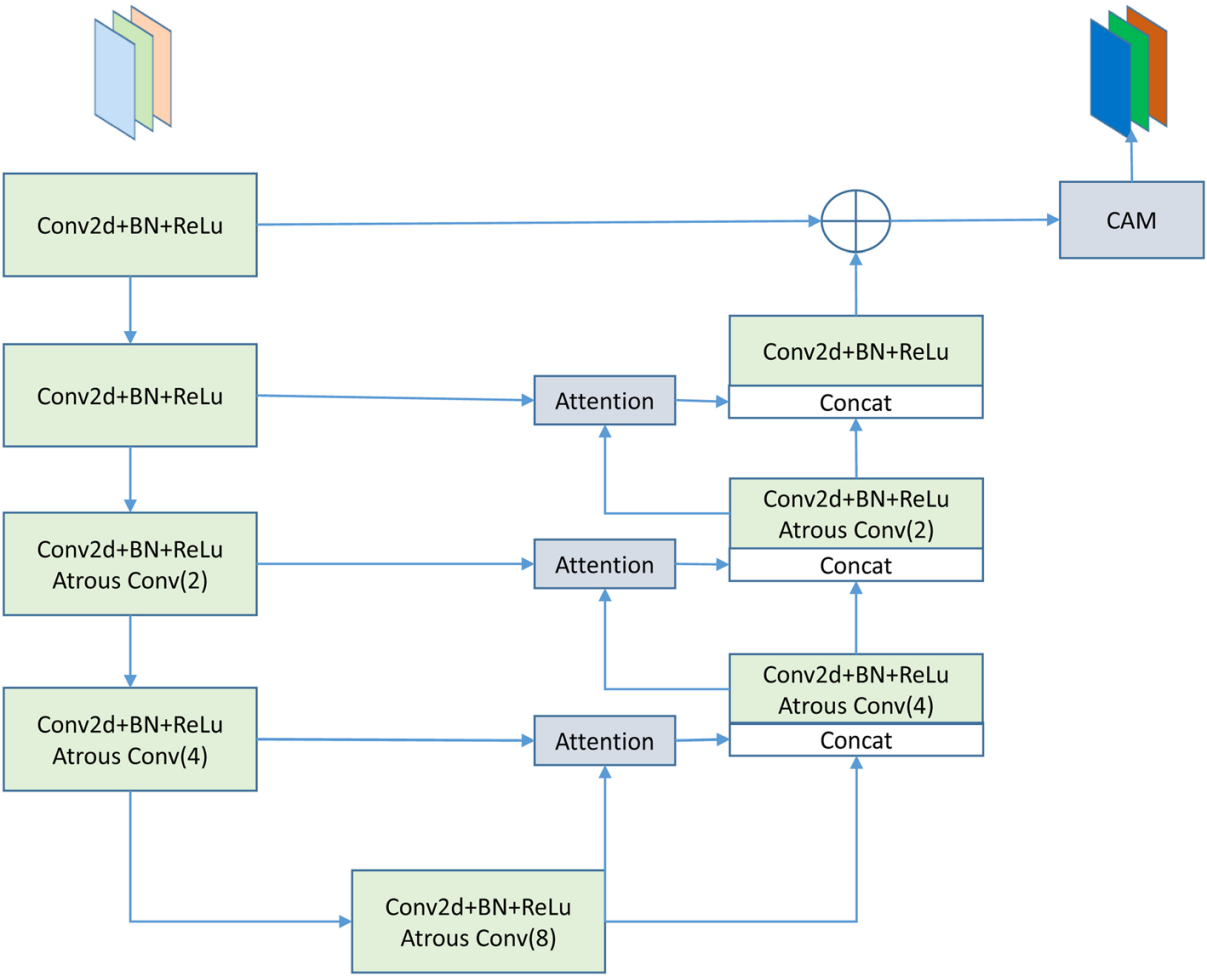
Architecture of DARSU-4F

As illustrated in Fig. 7, unlike other DARSU modules, since the input feature map undergoes five downsamplings, resulting in a significantly reduced resolution, continuing to downsample leads to the loss of some feature information. Therefore, in this module, dilated convolutions with dilation rates of 2, 4, and 8 are set at depths of 3–5 to expand the receptive field. Moreover, due to the low resolution at this layer and the scarcity of spatial feature information, extracting spatial attention is unnecessary. Thus, a CAM module is added to the final output to extract global salient feature information. The entire model maximizes the retention of partial and global salient area feature information with a minimal increase in computational cost, reducing information loss.

## Loss calculation

The loss function used in this paper combines Dice Loss and CrossEntropy Loss. Dice Loss is a common image segmentation loss function, with the following formula:

4 $$DiceLoss = 1 - \frac{{2\left| {X \cap Y} \right|}}{\left| X \right| + \left| Y \right|}$$

where $\frac{{2\left| {X \cap Y} \right|}}{\left| X \right| + \left| Y \right|}$ represents the Dice coefficient, which is primarily used to evaluate the similarity between two samples. In image segmentation tasks, $X$ represents the true labels, and $Y$ represents the predicted labels. $\left| {X \cap Y} \right|$ indicates the pixel values of the predicted labels in the true labels, and $\left| X \right| + \left| Y \right|$ represents the sum of the pixel values of the true and predicted labels. The specific calculation formula is as follows:

5 $$DiceLoss=1-\frac{2\sum_{i=1}^Ny_i{\widehat y}_i}{\sum_{i=1}^Ny_i+\sum_{i=1}^N{\widehat y}_1}$$

where $y_{i}$ is the label value of the pixel, $y_i$ is the predicted value of the pixel, and $N$ is the total number of pixels.

Owing to the loss saturation issue of Dice Loss, especially the saturation problem in segmenting detailed features, it is necessary to use CrossEntropy Loss to equally calculate the characteristics of each pixel, effectively complementing Dice Loss. The CrossEntropy Loss formula is as follows:

6 $$L = - \sum\nolimits_{i = 1}^{N} {y_{i} \log } y_{i}{\prime}$$

where $y_{i}$ is the label value of the pixel, $y_{i}{\prime}$ is the predicted value of the pixel.

## Experimental configurations

### Experimental parameter settings

The (DA-U)^2^Net system environment consists of a 16 vCPU Intel(R) Xeon(R) Platinum 8350C CPU @ 2. 60 GHz, paired with an RTX 3090 GPU (24 GB VRAM), and runs on Python 3.8, PyTorch 1.13.0, and CUDA 11.6. The Batchsize value is 2.The optimizer is Adam. The initial learning rate is 0.001.The weight decay is le^−4^.The number of training epochs is 100.

### Experimental data

#### Datasets

The DRIVE (Digital Retinal Images for Vessel Extraction) dataset was released in 2004 by the Image Sciences Institute, and features Dutch diabetic retinopathy data. This dataset includes 40 TIF color fundus images of size 565 × 584, with 40 GIF images manually annotated by two ophthalmologists, corresponding to 40 GIF mask images [27].

The CHASEDB1 (Challenging Database 1) dataset was released by Kingston University London, and features retinal image analysis data of 14 children’s left and right eyes. This dataset includes 28 JPG color fundus images of size 999 × 960, with 28 PNG images manually annotated by two ophthalmologists. CHASEDB1 does not come with corresponding mask images; we manually created the masks for this dataset [28].

The HRF (High-Resolution Fundus) dataset was released by the Ophthalmology Department at the University of California, San Diego (UCSD). This dataset includes 15 healthy fundus images, 15 diabetic retinopathy fundus images, and 15 glaucoma fundus images, with a resolution of 3504 × 2336 pixels. Each image corresponds to an expert manual segmentation result [29].

#### Preprocessing

**Fig. 8:**
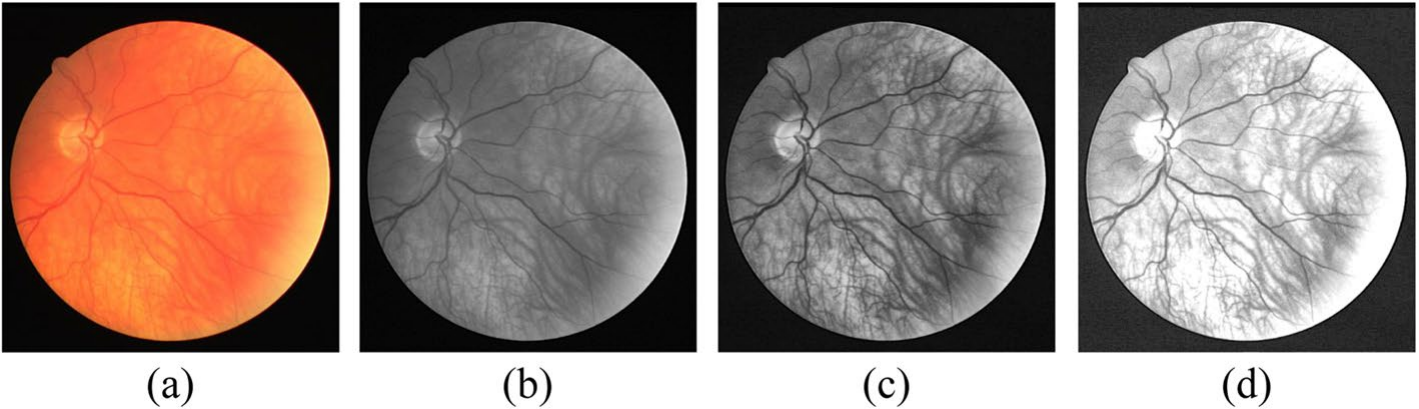
Typical images after each preprocessing step. **a** Original image; **b** G channel image; **c**Image after the CLAHE operation; **d** Image after the Gamma correction

Owing to the low contrast between the background and segmentation targets in fundus images and the presence of noise, preprocessing was conducted to improve the segmentation results. The preprocessing steps include the following:

- Image grayscale transformation through the G channel to reduce the contrast between the background and segmentation targets, followed by normalization [30];
- Enhancement of images through contrast-limited adaptive histogram equalization [31];
- Reduce the number of artifacts and improve of image information via gamma transformation. The preprocessed images after each preprocessing step are shown in Fig. 8.

**Fig. 9:**
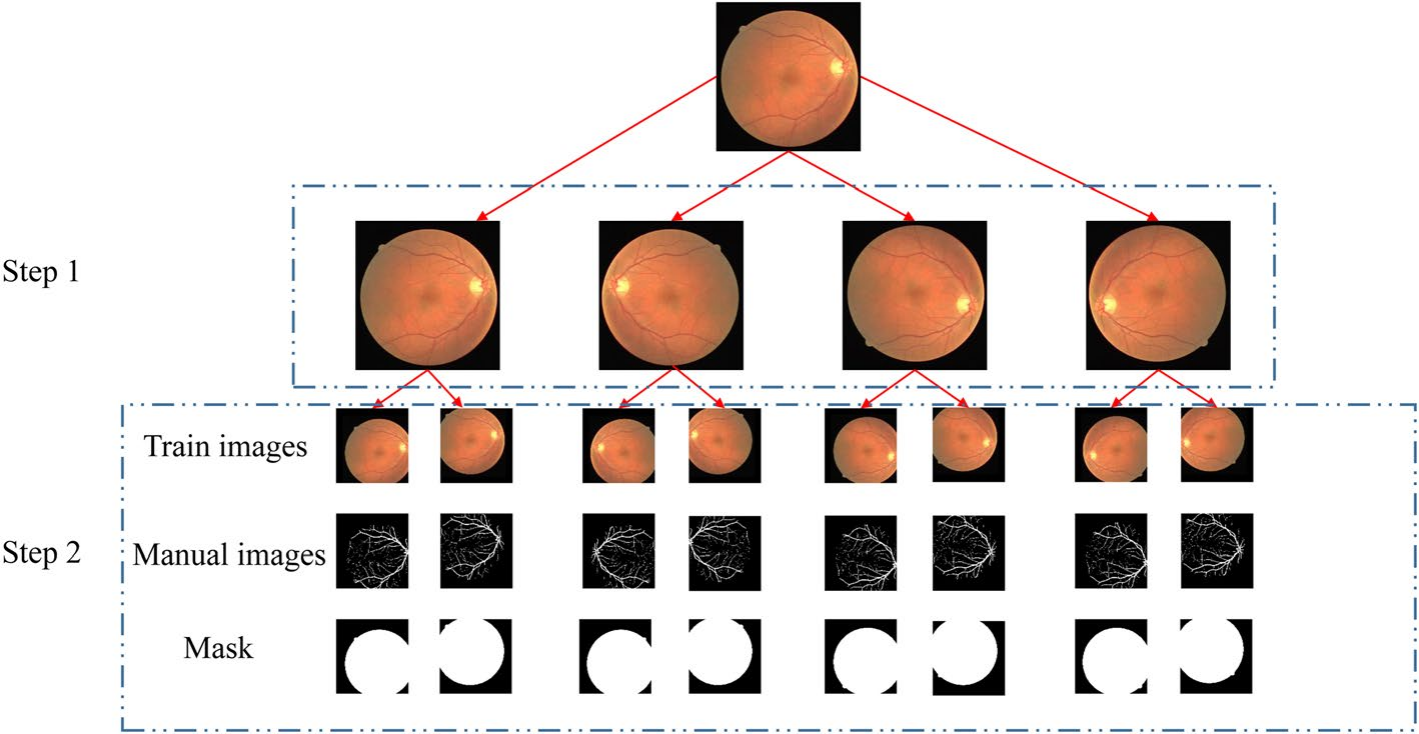
Process of data augmentation: Step 1. flipping images. Step 2. translating images. Includes: train images, manual images, and mask

#### Data augmentation

Due to the limited number of images in the DRIVE, CHASEDB1, and HRF datasets, there is a significant risk of overfitting during the training process. To address this, as shown in Fig. 9, images were initially augmented through horizontal flips, vertical flips, and combined horizontal and vertical flips, expanding the three datasets to 160, 112, and 144 images, respectively. Subsequently, the flipped images underwent horizontal and vertical translations with a translation coefficient of 50, ensuring that the integrity of the images was preserved. As a result, the DRIVE and CHASEDB1 datasets were further expanded to 800 and 560 images, respectively. However, due to the narrow margins on the left and right sides of the HRF dataset, horizontal and vertical translation augmentation was not applied.

Each dataset was divided into a training set and a testing set at a 4:1 ratio.

#### Evaluation metrics

This paper uses accuracy (Acc), specificity (SP), sensitivity (SE), the F1 score (FMeasure) [7] and Mean Intersection over Union (MIoU) [32] to evaluate and analyze the model.

## Results and discussion

### Comparison with existing methods

**Fig. 10:**
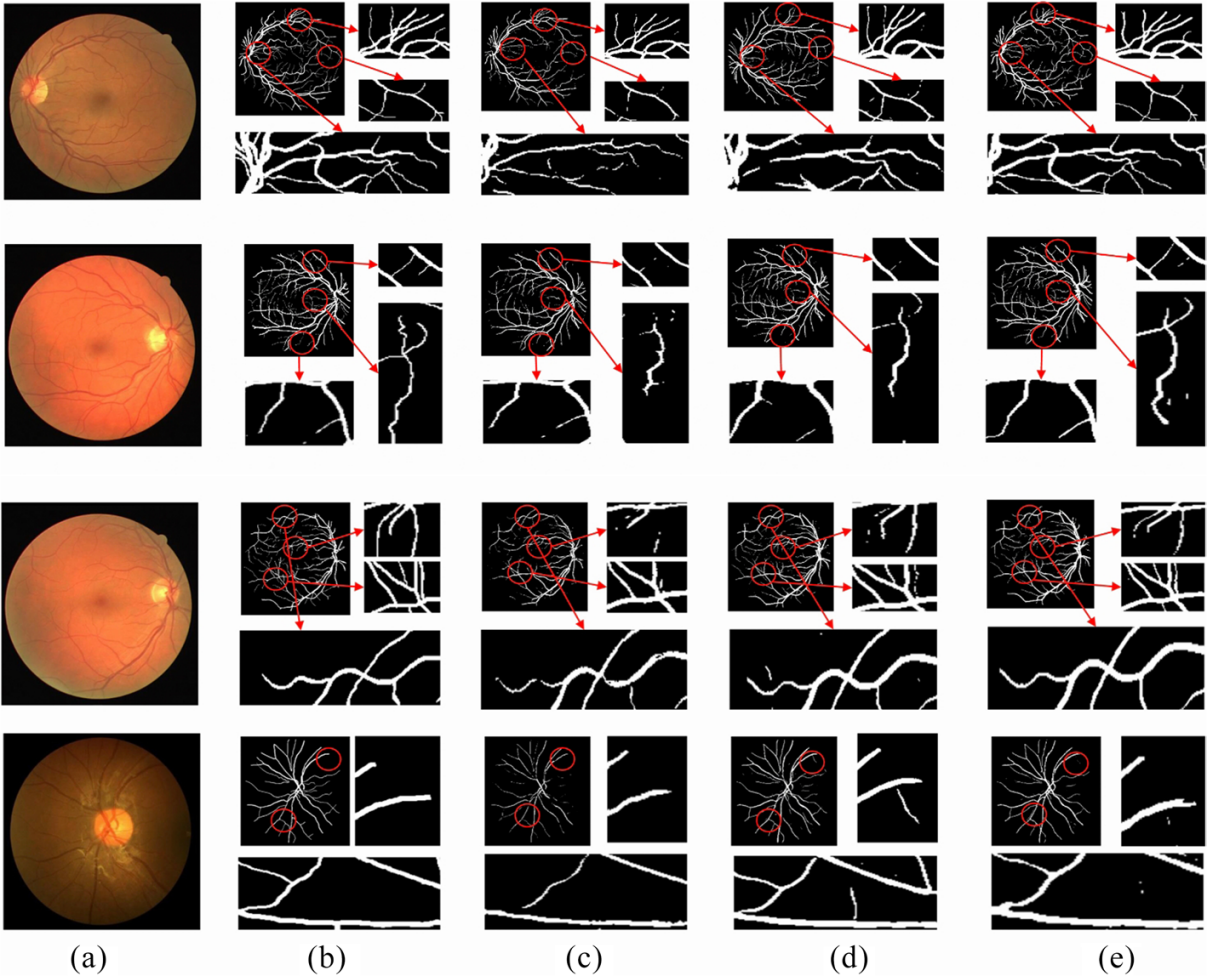
Segmentation results using the different methods on DRIVE and CHASEDB1. **a** Original image, **b** Ground truth, **c** U-Net, **d** R2UNet, **e** Ours

**Fig. 11:**
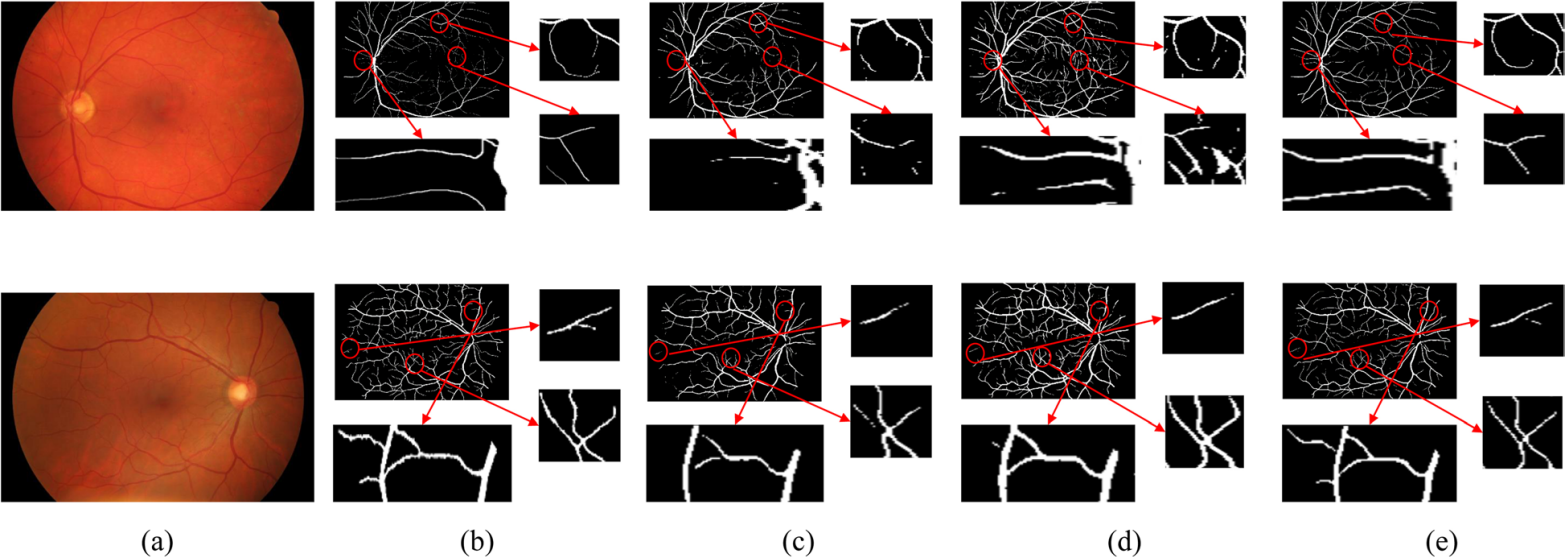
Segmentation results using the different methods on HRF. **a** Original image, **b** Ground truth, **c** U-Net, **d** R2UNet, **e** Ours

The proposed model was compared with the classic retinal segmentation model U-Net and the commonly improved U-Net model R2UNet [33] across three datasets: DRIVE, CHASEDB1, and HRF. The segmentation results are shown in Figs. 10 and 11.

Compared with the gold standard segmentation results (Figs. 10(b) and 11(b)), the proposed model achieves continuous and clear blood vessel structures, while the segmentation results of the other two models are relatively sparse. As shown in the first row of Fig. 10, fine blood vessels and complex regions were successfully segmented by the proposed model, which accurately restored the complex structures consistent with the gold standard. In contrast, the other two algorithms showed issues such as vessel breakage or redundant vessels, failing to fully replicate the real vascular structures. Moreover, as illustrated in Fig. 11, the proposed model produces smoother and clearer segmentation boundaries. This advantage facilitates disease diagnosis, especially in medical contexts where precise segmentation is critical.

A comparison of the segmentation performance metrics of U-Net and R2UNet on the DRIVE, CHASEDB1, and HRF datasets is shown in Table 1. For the DRIVE dataset, except for the sensitivity (SE) metric, which is lower than that of the UNet model, all other metrics surpass those of both the U-Net and R2UNet models. In the CHASEDB1 dataset, all metrics exceed those of the U-Net model, with both the global accuracy (Acc) and sensitivity (SE) metrics outperforming the R2UNet model. Although the other metrics are slightly lower than those of the U-Net model, they remain comparable. For the HRF dataset, except for the sensitivity (SE) metric, which is slightly lower than that of the R2UNet model, all other metrics show significant improvement. The HRF dataset has more detailed features compared to the other two datasets, and the notable increase in accuracy further confirms the superior performance of the proposed model.

**Table 1:** Comparative experimental data of U-Net and R2UNet

Database	Method	Acc	SP	SE	MIoU	F1
DRIVE	U-Net R2UNet	0.9477 0.9517	0.9540 0.9710	0.8900 0.8180	0.7819 0.8119	0.7660 0.8080
	Ours	**0.9546**	**0.9720**	**0.8300**	**0.8218**	**0.8200**
CHASEDB1	U-Net R2UNet	0.9476 0.9579	0.9490 **0.9750**	0.7810 0.7960	0.7223 **0.8011**	0.6660 **0.7860**
	Ours	**0.9581**	0.9720	**0.8160**	0.7970	0.7800
HRF	U-Net R2UNet	0.9505 0.9483	0.9750 0.9780	0.7140 0.7830	0.7620 0.7605	0.7320 0.7310
	Ours	**0.9640**	**0.9820**	0.7840	**0.8128**	**0.7990**

### Quantitative analysis of evaluation metrics compared with other state-of-the-art models

Tables 2, 3, and 4 present a comparison of the quantitative results of our model and existing methods based on evaluation metrics. The proposed model performs better than existing methods on the DRIVE and HRF datasets, with particularly significant improvements on HRF. However, its performance on the CHASEDB1 dataset is relatively weaker. Further analysis reveals that the DRIVE and HRF datasets are characterized by dense, fine vascular branches, while CHASEDB1 contains fewer and thicker vessels. This suggests that the proposed model is better suited for segmenting complex vascular structures but performs slightly less effectively on simpler fundus structures. The inferred reason is that the U^2^Net model is designed to handle more complex structures, making it more effective for multi-layered and numerous samples. However, for simpler samples with fewer structures, overfitting issues may lead to a decline in segmentation metrics.

**Table 2:** Quantitative result comparison of different models on the DRIVE

Method	Acc	SP	SE	F1
Ding et al. [34]	0.9537	0.9836	0.7487	0.8047
Azzopardi et al. [35]	0.9497	0.9710	0.7716	-
Xie et al. [36]	0.9524	0.9801	0.7627	0.8089
Fraz et al. [37]	0.9430	0.9768	0.7152	-
Hu et al. [38]	0.9533	0.9793	0.7772	-
Mo et al. [39]	0.9521	0.9780	0.7779	-
Shin et al. [40]	0.9271	0.9255	**0.9382**	-
Proposed method	**0.9546**	**0.9720**	0.8300	**0.8200**

**Table 3:** Quantitative result comparison of different models on the CHASEDB1

Method	Acc	SP	SE	F1
Ding et al. [34]	0.9566	0.9798	0.7475	0.7470
Azzopardi et al. [35]	0.9387	0.9587	0.7585	-
Xie et al. [36]	0.9597	0.9805	0.7516	**0.7815**
Fraz et al. [37]	0.9430	0.9768	0.7152	-
Mo et al. [39]	**0.9674**	**0.9844**	0.8147	-
Shin et al. [40]	0.9373	0.9364	**0.9598**	-
Yan et al. [41]	0.9607	0.9806	0.7641	-
Proposed method	0.9581	0.9720	0.8160	0.7800

**Table 4:** Quantitative result comparison of different models on the HRF

Method	Acc	SP	SE	F1
Shin et al. [40]	0.9349	0.9329	**0.9546**	-
Zhou et al. [42]	0.9559	0.9730	0.8310	**0.8211**
Cherukuri et al. [43]	0.9588	0.9733	0.8144	0.7832
Soomro et al. [44]	0.9620	0.9620	0.8290	-
Proposed method	**0.9640**	**0.9820**	0.7840	0.7990

### Ablation studies

To verify the effectiveness of the dual attention mechanism adopted in this paper, ablation studies were conducted on the DRIVE, CHASEDB1,and HRF datasets. The ablation study models include: 1) the Attention-U^2^Net model, which incorporates the channel attention mechanism into each RSU of the U^2^Net, forming the module A-RSU to replace the original RSU module; 2) The Attention-CBAM-U^2^Net model, which incorporates the CBAM attention mechanism’s SAM module into the ARSU-6, ARSU5, and ARSU-4 blocks of the Attention-U^2^Net model, and also introduces the CAM module into the ARSU-4F; 3) To better verify the effectiveness of the model, the CBAM-U^2^Net in this paper removes the channel attention mechanism between RSUs, retaining only the CBAM attention mechanism.

**Fig. 12:**
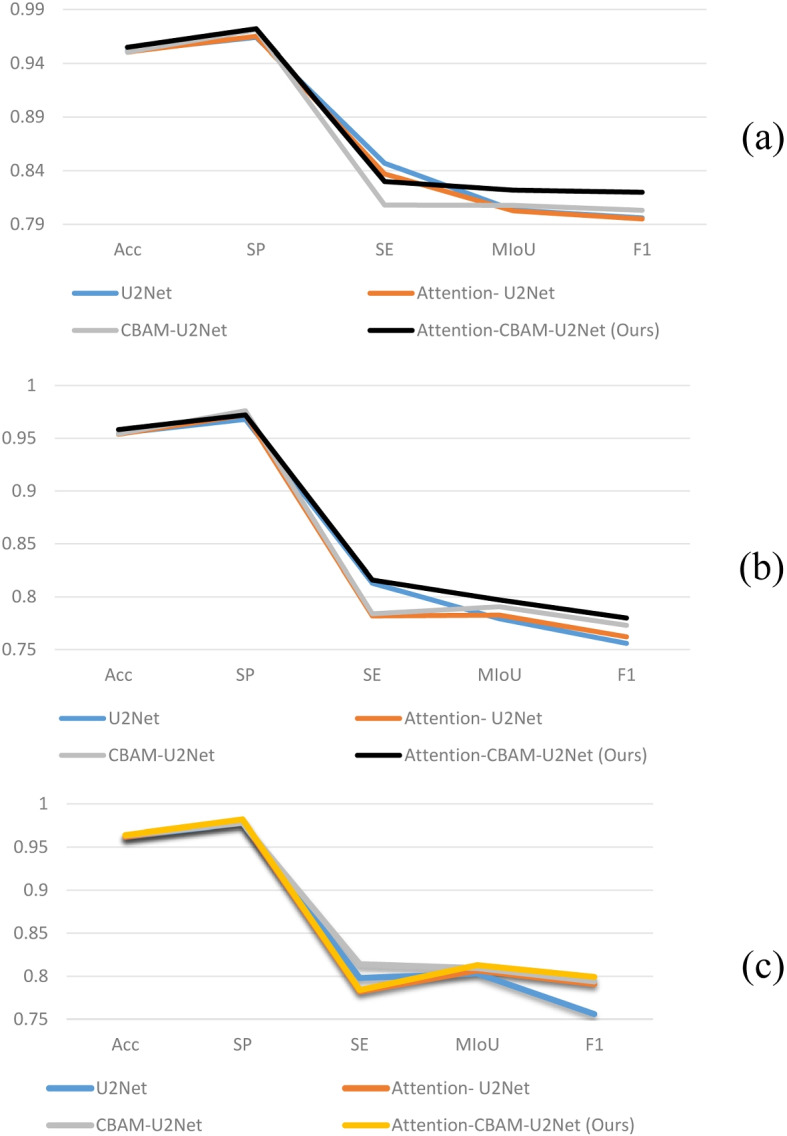
Comparison of the experimental performance of model Improvements on the DRIVE, CHASEDB1, and HRF. **a** DRIVE Dataset, **b** CHASEDB1 Dataset, **C** HRF Dataset

Tables 5, 6, 7, and Fig. 12 present a comparison of the experimental results between the original model and different module models. The results revealed that simply integrating the attention mechanism into the RSU module or adding the CBAM module does not effectively improve segmentation efficiency. However, after the dual attention mechanism is integrated, most metrics significantly improve. This confirms that while a hierarchical structure can capture rich features, it does not improve segmentation efficiency on its own. In contrast, a network model that incorporates a dual attention mechanism can better utilize the rich information characteristics of U^2^Net. The introduction of the attention mechanism enables the model to focus more on significant areas of space and feature information, suppress the expression of less important regions, and achieve an improvement in detail segmentation.

**Table 5:** Comparison of the experimental performance of the model improvements on the DRIVE dataset

U^2^Net	Attention	CBAM	Acc	SP	SE	MIoU	F1
✓			0.9510	0.9640	0.8470	0.8033	0.7960
✓	✓		0.9503	0.9650	**0.8370**	0.8025	0.7950
✓		✓	0.9502	0.9710	0.8080	0.8078	0.8030
✓	✓	✓	**0.9546**	**0.9720**	0.8300	**0.8218**	**0.8200**

**Table 6:** Comparison of the experimental performance of the model improvements on the CHASEDB1 Dataset

U^2^Net	Attention	CBAM	Acc	SP	SE	MIoU	F1
✓			0.9545	0.9680	0.8130	0.7792	0.7560
✓	✓		0.9537	0.9720	0.7820	0.7826	0.7620
✓		✓	0.9542	**0.9760**	0.7840	0.7905	0.7730
✓	✓	✓	**0.9581**	0.9720	**0.8160**	**0.7970**	**0.7800**

**Table 7:** Comparison of the experimental performance of the model improvements on the HRF Dataset

U^2^Net	Attention	CBAM	Acc	SP	SE	MIoU	F1
✓			0.9620	0.9780	0.7980	0.8031	0.7560
✓	✓		0.9620	0.9800	0.7820	0.8068	0.7910
✓		✓	0.9637	0.9780	**0.8140**	0.8094	0.7940
✓	✓	✓	**0.9640**	**0.9820**	0.78400	**0.8128**	**0.7990**

**Fig. 13:**
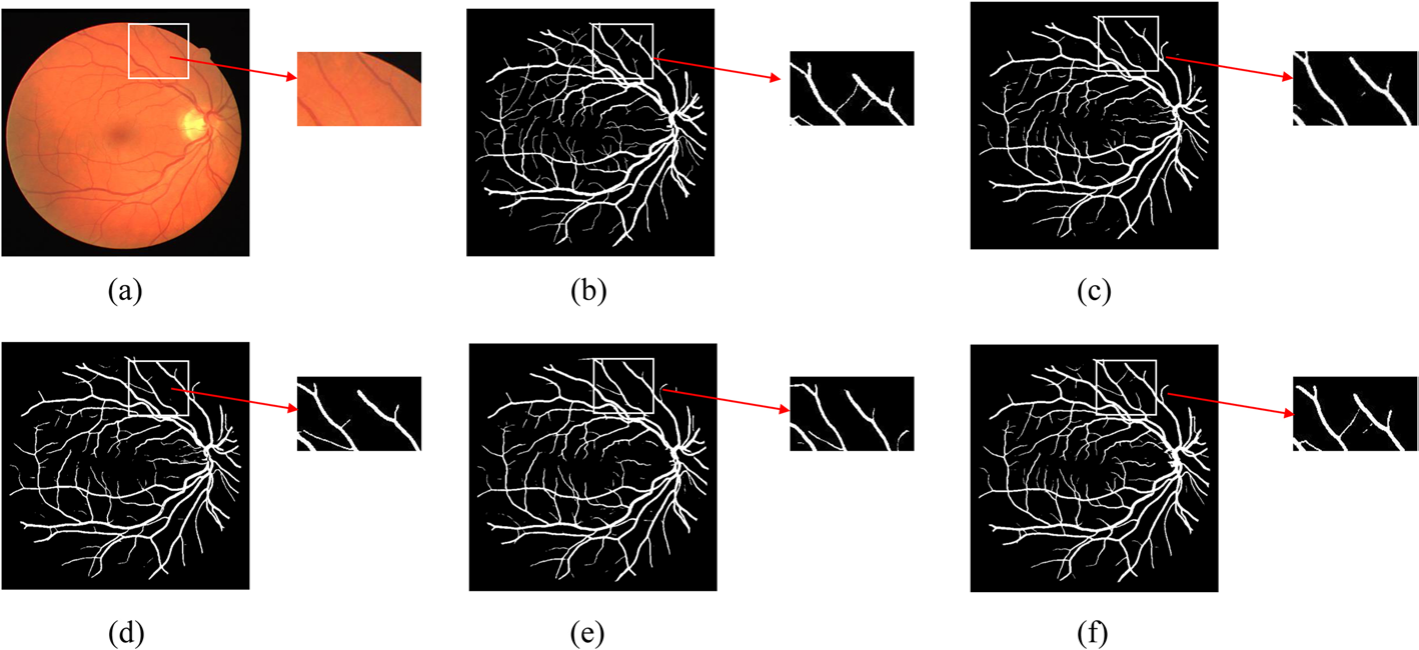
Detail Comparison in the Ablation Study on the DRIVE Dataset. **a** Original image, **b** Ground truth, **c** U-Net, **d** Attention-U^2^Net, **e** CBAMU^2^Net, **f** Attention-CBAM-U^2^Net

A comparison of the segmentation results of the ablation study models on the DRIVE, along with detailed segmentation slices, is shown in Fig. 13. The gold standard and our model reveal two finer capillaries within this area, whereas the other experimental models fail to detect these fine vessels in this location. Hence, the dual attention hierarchical model proposed in this paper demonstrates a superior ability to focus on detailed information, resulting in clearer segmentation outcomes.

**Fig. 14:**
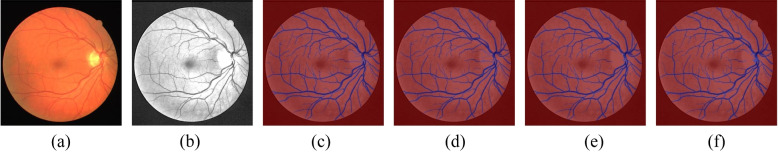
Heatmap Comparison on the DRIVE (**a**) Original image, **b** Detail image, **c** U-Net, **d** Attention-U^2^Net, **e** CBAM- U^2^Net, **f** Attention-CBAM-U^2^Net

To better demonstrate the effectiveness of the U-channel and spatial attention module and the U-channel dual attention module, we randomly selected an image from the DRIVE dataset for attention visualization in Fig. 14. Figure 14 illustrates: (a) the original image, (b) a grayscale image designed to more clearly display the vascular structures, (c) a model that lacks an attention mechanism, (d) a model that integrates only the channel attention mechanism, (e) a model that integrates only the CBAM attention mechanism, and (f) a model that integrates the dual attention mechanism. The heatmaps indicate that the model with dual attention focuses more effectively on the vascular regions.

## Discussion and evaluation

To demonstrate the model’s adaptability and reliability, this paper evaluates it on three clinical patient datasets: RBV, SMDG [45], and FIVES [46].

The Retina-Blood-Vessel (RBV) dataset consists of 100 high-resolution retinal fundus images captured using state-of-the-art imaging devices. Each image comes with pixel-level ground truth annotations that mark the exact locations of the blood vessels.These annotations facilitate the development and evaluation of advanced segmentation algorithms.

The Standardized Multi-channel Glaucoma Dataset (SMDG-19) is a collection of 19 publicly available datasets that have been standardized. It includes complete retinal fundus images for glaucoma, along with associated image metadata, such as optic disc, cup, and vessel segmentations, as well as any provided single-instance textual metadata (e.g., gender and age).This dataset is currently the largest publicly available repository of glaucoma retinal images.

The FIVES (Fundus Image Vessel Segmentation) dataset contains 800 highresolution color retinal images, each manually annotated pixel-by-pixel through standardized crowdsourcing by medical professionals. The data was sourced from 573 patients seen at the Ophthalmology Center of the Second Affiliated Hospital of Zhejiang University (SHAZU) between 2016 and 2021, with ages ranging from 4 to 83 years. The dataset contains a significant number of low-quality images, some of which are nearly impossible to distinguish with the naked eye due to imaging issues.Effective segmentation of these images requires clinical experience, which makes this dataset valuable for demonstrating the model’s adaptability and reliability.

After performing transfer learning with the proposed model across the three datasets, its performance was evaluated, with the results shown in Table 8. The data indicates a significant improvement across all metrics compared to public datasets, with especially strong performance on the SMDG and FIVES datasets. The accuracy (ACC) was 0.9871, specificity (SP) was 0.9930, sensitivity (SE) was 0.9220, mean intersection over union (MIoU) was 0.8989,and the F1 score was 0.8960, demonstrating the model’s significant advantages in segmenting large-scale, complex clinical datasets.

**Table 8:** Comparative Experimental Results for RBV, SMDG, and FIVES

Data	Acc	SP	SE	MIoU	F1
RBV	0.9715	0.9830	0.7490	0.7694	0.7250
FIVES	0.9689	0.9910	**0.9220**	**0.8989**	**0.8960**
SMDG	**0.9871**	**0.9930**	0.8650	0.8750	0.8660

**Fig. 15:**
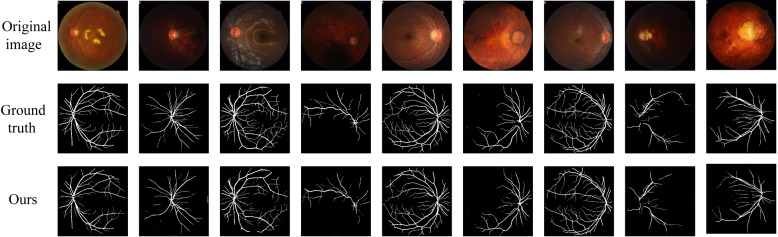
Segmentation results of the proposed model on low-quality data

The FIVES dataset contains a large number of low-quality images, some of which are nearly indistinguishable due to capture issues. Clinical doctors must have extensive experience to achieve effective segmentation. As shown in the comparison in Fig. 15, the proposed model demonstrates superior performance even on low-quality images, with segmentation results closely aligned with the ground truth, indicating that its segmentation capability is on par with experienced clinicians.

The evaluation results on clinically challenging datasets provide stronger evidence of the model’s robustness and its ability to generalize across datasets, ensuring its applicability in real-world scenarios.

## Conclusion

This paper proposes the integration of multi-attention mechanisms into the RSU submodules of the U^2^Net for retinal segmentation tasks. The proposed (DA-U)^2^Net effectively captures multiscale contextual information and promotes the fusion of features at different levels to obtain richer semantic feature representations. Specifically, for different RSU modules, the spatial attention module (SAM) is integrated into the outputs of higher-resolution level modules, achieving dual attention in both spatial and channel dimensions, which effectively extracts multiscale contextual information. In lower-resolution level modules, due to the scarcity of spatial information, the channel attention module (CAM) is integrated to prevent detail loss while simultaneously suppressing background noise. The model effectively utilizes the hierarchical network characteristics of U^2^Net to extract rich image information features. The integration of the dual attention mechanism enables the model to effectively suppress noise expression, while the combination of spatial and channel attention reduces the loss of key information, ultimately improving segmentation accuracy. Extensive comparative evaluations on three publicly available retinal fundus extended databases demonstrate the superiority of the proposed method over most existing approaches. Finally, the evaluation on three clinically challenging datasets shows a significant improvement across all metrics, particularly in the segmentation of low-quality images, which is close to the ground truth. This proves the model’s applicability in real-world settings.

The proposed model, however, has the following issues: 1)The structure of the model is complex, leading to high computational costs; 2)The complexity of the model structure imposes high demands on the quantity and quality of images, which is particularly evident in its performance on high-resolution HRF datasets compared to other datasets; 3)While the model achieves more precise segmentation for images rich in details, its segmentation performance is poorer for images with sparse vascular distributions.

These points represent areas for future research improvement. Nonetheless, the proposed model introduces a novel stereoscopic architecture integrated with multiple attention mechanisms, paving the way for complex ocular vascular segmentation. It is believed that the model can be readily extended to other medical image segmentation tasks, particularly those facing significant challenges of large-scale variations and complex features.

## Data Availability

The datasets used in this study are available in the DRIVE Dataset, which can be accessed at https://drive.grand-challenge.org/, and the CHASEDB1 Dataset, accessible at https://researchdata.kingston.ac.uk/96/.
